# Skin Barrier Protection

**DOI:** 10.1155/2012/691954

**Published:** 2012-12-23

**Authors:** Georgios N. Stamatas, Alex Zvulunov, Paul Horowitz, Gary L. Grove

**Affiliations:** ^1^Skin Care R&D, Johnson & Johnson Santé Beauté France, 92787 Issy-les-Moulineaux, France; ^2^Faculty of Health Sciences, Ben Gurion University and Soroka Medical Center, 84101 Beer Sheva, Israel; ^3^Discovery Pediatrics Inc., Valencia, CA 91355, USA; ^4^cyberDERM Inc., Broomall, PA 19008, USA

The largest organ of the human body is the skin with its ~2 m^2^ surface area that envelopes the whole organism defining its physical border and which most importantly provides a barrier against internal organ dehydration and external penetration of noxious substances. The critical importance of the skin barrier is appreciated when it is lost such as in skin burns or compromised such as in atopic dermatitis, affecting the overall wellbeing of the individual. Moreover, it has been shown that the skin barrier function undergoes a maturation process during the first years of life. It is therefore of interest to identify skin care routines such as washing and bathing that would not be damaging to the skin barrier and if possible enhancing its protection. Furthermore, barrier enhancement aids in the prevention and treatment of certain conditions such as atopic dermatitis. Recent scientific discoveries in skin biology and formulation science have advanced the understanding of the regulatory mechanisms that control skin barrier homeostasis as well as our knowledge of skin-product interactions. Application of this knowledge has led to the design of appropriate skin care products and the design of tests that can demonstrate barrier-related benefits. This special issue of the Dermatology Research and Practice addresses these issues.

The paper by L. Telofski et al. titled “*The infant skin barrier: can we preserve, protect, and enhance the barrier?*” provides an introduction to the recent findings on skin barrier maturation after birth that lasts for the first few years of life. The authors present on healthy skin barrier development as well as problems that can arise during infancy related to abnormal skin conditions and barrier integrity. They then discuss appropriate cleansing routines that should respect the distinct nature of infant skin and the use of emollients to protect and enhance the infant skin barrier function.

In the paper titled “*Management of patients with atopic dermatitis: the role of emollient therapy*” by M. Mack Correa and J. Nebus, the authors discuss in more detail the use of emollients as baseline support therapy to the use of corticosteroids or calcineurin inhibitors. Prevention strategies are also presented and include appropriate skin care routines, avoiding allergic triggers, and regular use of emollients to improve the skin barrier function. Y. Valdman-Grinshpoun et al. present the dermatologist's point of view in treating and managing atopic dermatitis in their contribution titled “*Barrier-restoring therapies in atopic dermatitis: current approaches and future perspectives*.” This paper makes the link between skin barrier dysfunction and atopic dermatitis. It goes on to discuss the importance of skin barrier therapy approaches for its management including the use of corticosteroids and immunomodulators, as well as the potential requirement of short-term topical or systemic use of antibiotics in cases of infected lesions.

Another barrier-related disease, contact dermatitis, is reviewed in the paper by Y. Yoshihisa and T. Shimizu titled “*Metal allergy and systemic contact dermatitis: an overview*.” Known metals common in our environment, such as nickel, cobalt, zinc, and chromium can result in allergic contact dermatitis. The authors present *in vivo* and *in vitro* diagnostic tests of metal sensitivity.

Other aspects of skin barrier protection are covered by the following two papers: “*Cleansing formulations that respect skin barrier integrity*” by R. Walters et al. and “*Methods to assess the protective efficacy of emollients against climatic and chemical aggressors*” by R. Roure et al. The former presents information about innovations in mild surfactant technologies used in cleansing products. As cleansing is an everyday activity that brings these products in contact with the skin, it is important to understand what makes a surfactant potentially aggressive to the lipids of the stratum corneum that provide a large part of the skin's barrier function. The use of hydrophobically modified polymers as surfactants in skin cleansing products is being introduced to enhance the stability and size of micelles and therefore the mildness to the skin. The latter mentioned paper deals with *in vivo* skin protocols that are being used to assess the protective action of emollients on the skin. Such models simulate skin exposure to cold wind or to an irritant such as sodium lauryl sulfate. Data are then presented to demonstrate how these investigative methods can be used to show how exposures can impact skin barrier and skin protection.

*Georgios N. Stamatas* *Georgios N. Stamatas* *Alex Zvulunov* *Alex Zvulunov* *Paul Horowitz* *Paul Horowitz* *Gary L. Grove* *Gary L. Grove*

